# The Investigation of the Chemical Composition and Applicability of Gold Nanoparticles Synthesized with *Amygdalus communis* (Almond) Leaf Aqueous Extract as Antimicrobial and Anticancer Agents

**DOI:** 10.3390/molecules28062428

**Published:** 2023-03-07

**Authors:** Mehmet Fırat Baran, Cumali Keskin, Ayşe Baran, Aziz Eftekhari, Sabina Omarova, Rovshan Khalilov, Mehmet Tevfik Adican, Gvozden Rosić, Dragica Selakovic, Mahmut Yıldıztekin, Kadri Kurt, Canan Aytuğ Ava, Mehmet Nuri Atalar

**Affiliations:** 1 Department of Food Technology, Vocational School of Technical Sciences, Batman University, Batman 72100, Turkey; 2 Department of Medical Services and Techniques, Vocational School of Health Services, Mardin Artuklu University, Mardin 47000, Turkey; 3 Department of Biology, Graduate Education Institute, Mardin Artuklu University, Mardin 47000, Turkey; 4 Department of Biochemistry, Faculty of Science, Ege University, Izmir 35040, Turkey; 5 Institute of Molecular Biology & Biotechnologies, Ministry of Science and Education Republic of Azerbaijan, 11 Izzat Nabiyev, Baku AZ1073, Azerbaijan; 6 Department of Biophysics and Biochemistry, Baku State University, Baku AZ1148, Azerbaijan; 7 Department of Electricity and Energy, Vocational School, Mardin Artuklu University, Mardin 47000, Turkey; 8 Department of Physiology, Faculty of Medical Sciences, University of Kragujevac, 34000 Kragujevac, Serbia; 9 Department of Electricity and Energy, Beşiri OSB Vocational School, Batman University, Batman 72100, Turkey; 10 Department of Medical Services and Techniques, Koycegiz Vocational School of Health Services, Mugla Sitki Kocman University, Mugla 48170, Turkey; 11 Dicle University Science and Technology Application and Research Center, Dicle University, Diyarbakır 21280, Turkey; 12 Department of Nutrition and Dietetics, Faculty of Health Sciences, Iğdır University, Iğdır 76000, Turkey

**Keywords:** *Amygdalus communis* L., anticancer, antimicrobial, AuNPs, green nanotechnology, chemical composition, nanomedicine

## Abstract

The current work’s main objective was to determine the chemical composition of *Amygdalus communis* (AC) leaf extract and examine the antibacterial and cytotoxic properties of biosynthesized gold nanoparticles (AuNPs). The chemical composition of AC leaf extract was determined using LC-ESI/MS/MS to detect compounds that may be responsible for the reducing, stabilizing, and capping steps in the synthesis of nanoparticles and their biological activities. The AC-AuNPs were spherical, with a particle size lower than 100 nm and a face-centered cubic structure. The EDX spectrum confirmed the formation of AuNPs and a negative zeta potential value (−27.7 mV) suggested their physicochemical stability. The in vitro cytotoxic efficacy of the AC-AuNPs against colorectal adenocarcinoma (Caco-2), glioma (U118), and ovarian (Skov-3) cancer cell lines and human dermal fibroblasts (HDFs) was evaluated by MTT assay. CaCo-2 cell proliferation was effectively inhibited by the AC-AuNPs at concentrations between 25 and 100 g mL^−1^. The AC-AuNPs exerted preeminent antimicrobial activity against *Bacillus subtilis* with an MIC of 0.02 μg/mL, whilst good activity was shown against *Staphylococcus aureus* bacteria and *Candida albicans* yeast with an MIC of 0.12 μg/mL. Ultimately, the results support the high antibacterial and anticancer potential of biosynthesized AuNPs from AC leaf extract.

## 1. Introduction

Metallic nanoparticles are a topic of research that attracts a lot of attention in the field of nanoscience. They hold great promise for biological applications due to their controlled geometry, optics, and surface chemistry [1]. These innovations, which have the potential for further growth, benefit every facet of human existence and are gaining greater attention in the fields of medicine and life sciences [2].

AuNPs and AgNPs, among other metal nanoparticles, have recently received a lot of interest. AuNPs have a variety of uses, including sensory probes, medication administration, diagnostics, cancer therapy, and catalysis [3]. Gold colloids are widely used in chemistry, biology, engineering, and medicine and have interested scientists for more than a century [4]. Monodisperse gold nanoparticles with a diameter of 1 to 3 nm may be created using the Brust-Schiffrin process [5]. Nanoparticles are frequently employed for medication delivery because they have high in vivo stability and cell absorption efficiency. Additionally, metal/metal oxide nanoparticles can accelerate the oxidation of superoxide anions and hydrogen peroxide and mimic the action of antioxidant enzymes [6]. Metal ions are released during the breakdown of metal/metal oxide nanoparticles, which reduces inflammation [7]. Gold nanoparticles, nanoclusters, nanocages, nanorods, nanostars, nanoshells, and nanoplates are examples of gold nanomaterials [8]. As photothermal treatment agents that target malignant cells with antibody-coated surfaces, gold nanorods and nanoshells coupled with antibodies are currently being researched. An important family of materials known as gold nanoconjugates has already shown promise in applications related to fundamental cell biology [9]. Gold nanoconjugates may be used to introduce compounds into cells very effectively. Numerous independent research teams have examined the toxicity of different types and dimensions of gold nanoconjugates [4]. Liposomes and polymer particles are substantially larger than gold nanoparticles (AuNPs), which are much smaller [10]. In addition, AuNPs inherently have distinct pro-oxidant and anti-inflammatory effects in comparison to other NPs [11]. On the one hand, GNPs help to produce reactive oxygen species (ROS), an efficient reagent in the treatment of cancer [12].

Several methods can be used to produce AuNPs. Due to its advantages over other approaches, such as being simple, ecologically safe, non-detrimental to human health, and its ability to produce more products as well as those having biocompatible architectures, research on green synthesis using plant resources is gaining greater interest. Physical and chemical methods are costly, involve time-consuming synthesis processes, and use compounds that are potentially harmful to both people and the environment [13]. By reducing ionized gold in the aqueous medium, the functional groups of phytochemicals (such as alcohols, phenols, or amines) in plant source extracts play a crucial part in the production of nanoparticles in synthesis studies [14].

The *Prunus* species *Amygdalus communis* L., a member of the Rosaceae family, grows in subtropical areas, especially in the Mediterranean region, southwest Asia, and the Middle East. *Prunus communis* L., *Prunus amygdalus* Batsch, and *Prunus dulcis* are some of the numerous names for *A. communis* L. (Miller). The almond kernel is responsible for the nutritiousness of almonds [15]. Almond seeds are eaten as a snack or added to processed foods such as chocolates, confections, and many types of baked products. As opposed to their edible counterparts, the inedible parts, such as the hull, shell, skin, and other aerial parts of leaves and stems, are discarded or utilized as fuel or animal feed [16].

Recent studies have linked almonds to a range of health advantages, including their potent free radical scavenging abilities, hepatoprotective properties, anti-diabetic effects, cholesterol-lowering effects, as well as antiviral and anticancer (human prostate cancer cell lines [PC3, Du 145], human ductal epithelial breast tumor cell line [T47D], human pancreatic cancer cells [PANC-1], human leukemia cells [K562], and human malignant melanoma cell line [A375.S2]) activities [17,18,19,20].

Phenolic acids and flavonoids have been identified in the kernels and skins of almonds through phytochemical research. Triterpenes, chlorogenic acid derivatives, and flavonoids have also been isolated in the shells. The bioactive substances in almond tree leaves, such as alcohol or phenolic groups, have high potential but the leaves are discarded as agricultural waste. More research has revealed the abundance of tannins, phenols, flavonoids, carotenoids, and alkaloids in the *Amygdalus* species [21,22]. It has also been noted that these compounds have high antioxidant activity and can be regarded as playing a significant role in the reduction of gold to AuNPs.

*A. communis* leaf aqueous extract was used in this work to create gold nanoparticles (AuNPs), which were then characterized and their potential antibacterial and anticancer properties were examined. A more economical and ecologically responsible alternative to conventional methods for the creation of biocompatible nanoparticles is provided by this unique green synthesis technique. The synthesized green nanomaterials may potentially have several uses in the pharmaceutical sector, including the production of useful nanodevices and creation of innovative medications.

## 2. Results and Discussion

### 2.1. Chemical Composition

**Figure 1:**
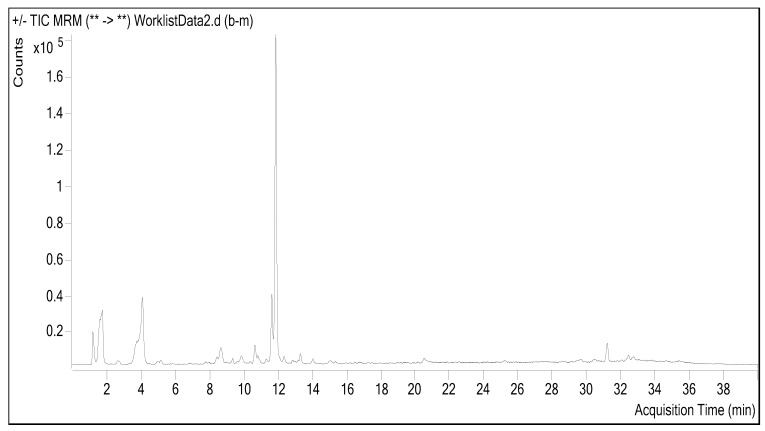
Sample chromatogram from LC-ESI-MS/MS analysis.

The chemical composition of *A. communis* leaf extract was evaluated using LC-ESI-MS/MS analysis. A standard sample chromatogram from LC-ESI-MS/MS analysis is shown in Figure 1. In the investigation, which tested 53 phenolic and flavonoid standards, 16 components were discovered (Table 1). The chemical profiling of the crude extract of *A. communis* leaves revealed the presence of a variety of phytochemicals, predominantly phenolics, resulting in the identification of eight major high-feature compounds: two major phenolic acids (shikimic and gallic acids), one beta hydroxy acid (salicylic acid); one polyphenol (polydatine), and tree flavonol glucosides (quercimeritrin, hyperoside, quercetin-3-glucoside, and rutin). The components with the highest concentrations were shikimic acid, rutin, gallic acid, quercetin-3-glucoside, quersimeritrin, and hyperoside, respectively. In the anabolic process of hydroaromatic chemicals, shikimic acid (SA) is a crucial step. In the chemical synthesis of oseltamivir (Tamiflu^®^), an antiviral medication, SA, a hydroaromatic natural substance, serves as a chiral precursor. SA may also be combined to form several bioactive substances, including zeylenone (an antitumor compound).

The biological activities of the AC extract that have been examined may be attributed to the incredibly high concentration of shikimic acid [23]. Furthermore, salicylic acid, protocatechuic acid, and polydatine were found as important chemicals that may be responsible for their biological features. Polydatine, also known as Piceid, is a monocrystalline natural precursor and glycoside form of resveratrol. Polydatine, which is isolated from the bark of *Picea sitchensis* or *Polygonum cuspidatum*, has been found in grapes, peanuts, hop cones, red wine, hop pellets, cocoa-containing goods, chocolate products, and a variety of daily foods. Polydatine has anti-inflammatory, immunoregulatory, antioxidant, and anti-tumor properties. It has been demonstrated to have cytotoxic effects against colorectal cancer cells by causing cell inhibition and apoptosis [24,25].

Rutin is a specific antioxidant flavonoid found mostly in fruits, vegetables, grains, and a variety of other plant-based human foods. Rutin has been shown to suppress the growth of breast, colon, lung, and prostate cancers, other malignancies. Furthermore, rutin promotes apoptosis in conjunction with medicinal drugs. Combining rutin with other chemotherapy medications can also reduce medication resistance and the adverse effects of chemotherapy [26].

### 2.2. Characterization of Biogenic AuNPs

#### 2.2.1. UV-Vis Spectrum Data of AC-AuNPs

**Figure 2:**
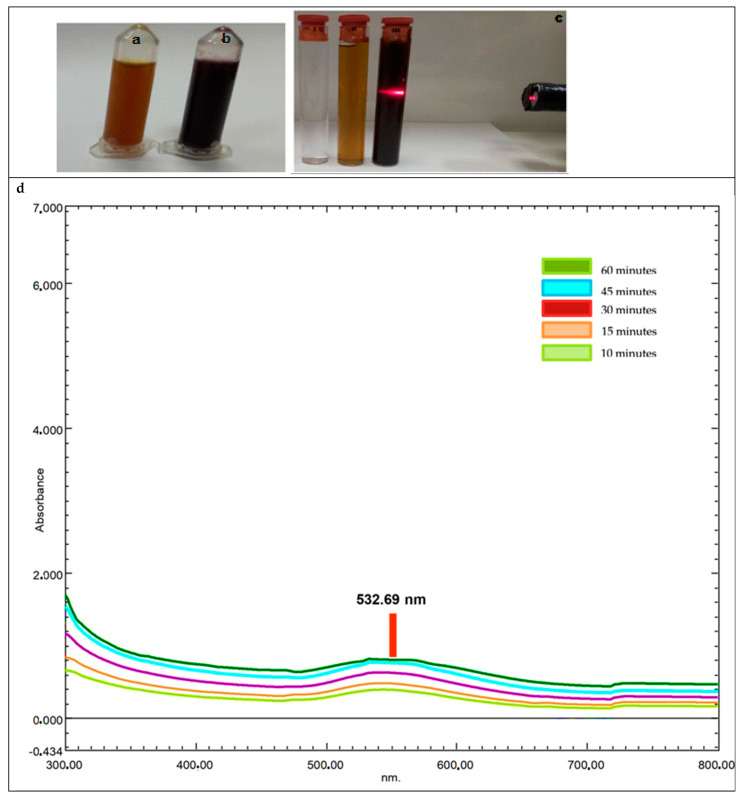
(**a**) *Amygdalus communis* leaf extract. (**b**) Color change due to the formation of synthesized AC-AuNPs. (**c**) The presence of AuNPs in colloidal form confirmed by the Tyndall effect against the laser beam (I. HAuCl_4_ solution, II. plant extract, and III. colored liquid formed as a result of synthesis). (**d**) Time-dependent maximum absorbances (10 to 60 min).

A pink-red color shift was seen 60 min after mixing the HAuCl_4_ solution and AC leaf extract. Peaks in surface plasmon resonance (SPR) occur when metal nanoparticles are produced. UV-visible spectroscopy was used to identify the distinctive gold nanoparticle SPR pattern (Figure 2). The time-dependent (10–60 min) change in the UV-Vis spectrum of gold nanoparticles synthesized from an aqueous leaf extract of AC using 25 mM HAuCl_4_ as the gold precursor is shown in Figure 2. The prominent SPR band that appears at 532.7 nm when 30 mL of the plant extract was combined with 120 mL of tetra chloroauric (III) acid solution (25 mM) confirmed the synthesis of AuNPs (Figure 2d). In Figure 2c, the Tyndall effect was observed, as a macroscopic observation confirming the presence of a colloidal system with the formation of the AC-AuNPs as a result of synthesis [27,28].

#### 2.2.2. X-ray Diffraction Analysis Data

**Figure 3:**
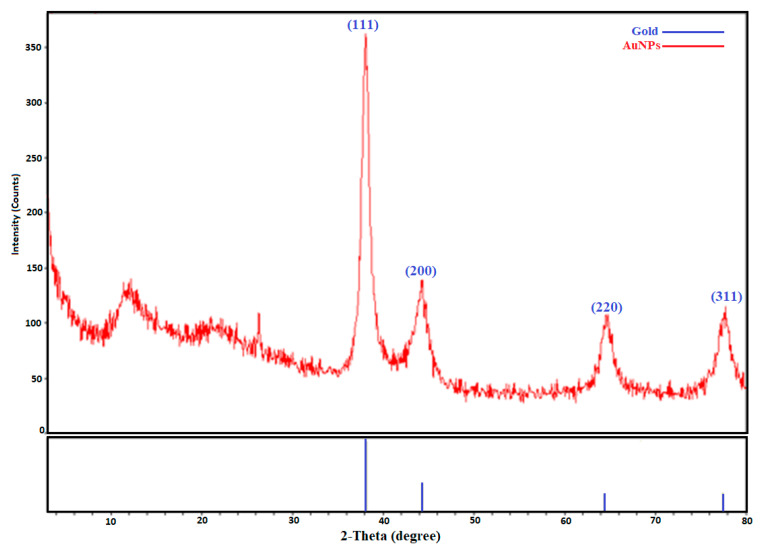
X-ray diffractogram of biogenic gold nanoparticles.

The crystalline nature and elemental content of the produced nanoparticles were determined using X-ray diffraction (XRD) (Figure 3). The face-centered cubic crystalline (FCC) structure of the gold nanoparticles was determined via the XRD spectrum, which revealed four diffraction bands at 2theta = 38.10°, 44.40°, 64.80°, and 77.86°. These were the typical Bragg’s diffraction planes (111°, 200°, 220°, and 310°) of the AuNPs (Figure 2). Our findings were consistent with previous reports [29,30]. The presence of small weak peaks that did not affect the peak width next to the peaks of high wide-angle Bragg angles indicated that phytochemicals in organic form played a role in stability. Thus, it was obvious from the XRD pattern that the Au nanoparticles generated via the reduction of AuCl_4_ ions by the aqueous extract of *A. communis* leaves were crystalline in form. The widening of the Bragg peaks was an indication of the existence of nanoparticles. Using the Debye-equation Scherrer’s in Formula (1) and the width of the (111°) Bragg reflection, the mean particle size was calculated to be 24.3 nm, which was in agreement with the FESEM findings.

#### 2.2.3. FTIR Spectroscopy Data

**Figure 4:**
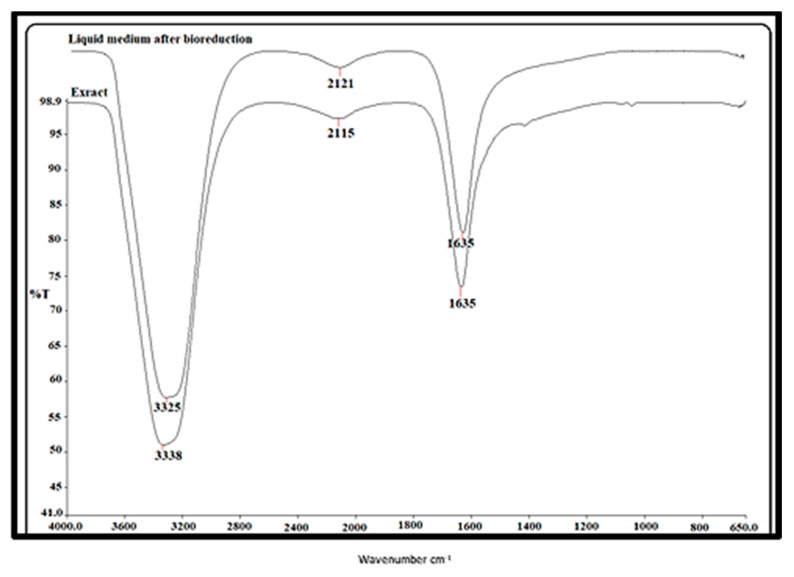
FTIR spectra of *Amygdalus communis* leaf extract and biogenic functionalized gold nanoparticles.

The various functions involved in the reduction of metal-to-metal nanoparticles were examined using FTIR analysis. Figure 4 displays the IR spectra of the biogenic gold nanoparticles and *Amygdalus communis* leaf extract. The FTIR spectrum of the gold nanoparticles produced by the plant extract is represented in Figure 4. Three prominent bands at wave numbers 3337.93–3324.68, 2120.51–2115.25, and 1635.12–1634.97 cm^−1^ that showed some shift in the corresponding gold nanoparticles were observed in the aqueous extract of *Amygdalus communis* and liquid medium (after bioreduction) (Figure 4). These peaks may have been attributable to the ester bonds in polyphenolic compounds, the OH-stretching vibration of phenolic compounds, and the carbonyl group stretching vibration, respectively. The O–H, alkyne (–C=C–), and amine (–NO) groups produced by the bioreduction of +3 valent Au metal in the aqueous medium to form 0 valence AuNPs demonstrated that they could be responsible for the stability [31,32,33].

#### 2.2.4. EDX Pattern of Biogenic Gold Nanoparticles

**Figure 5:**
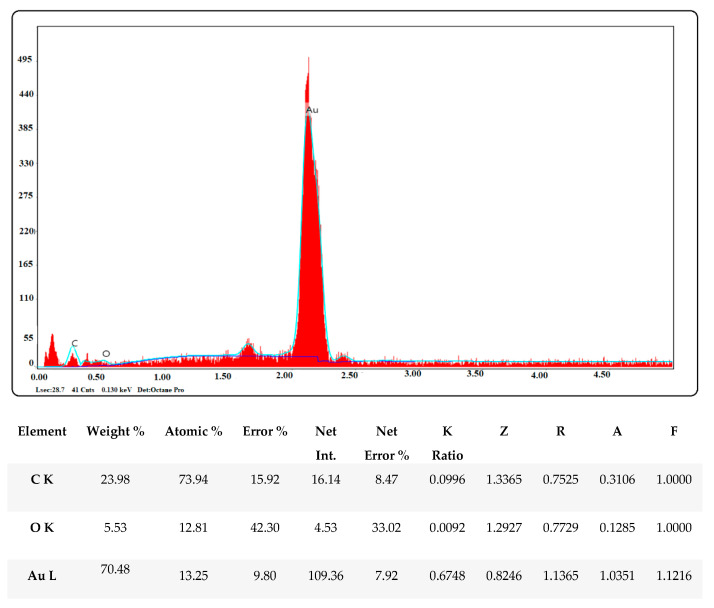
Elemental profile (EDX) of gold nanoparticle synthesis with *Amygdalus communis* leaf extract.

The EDX spectrum of the synthesized gold nanoparticles showed a prominent band of approximately 2–2.5 keV, showing that elemental gold was a significant constituent of the gold nanoparticles. At 0.2 and 0.5 keV, respectively, some faint signals from C and O were also observed. These signals might have originated from the capping biomolecules and carbon-supported gold grid that were utilized to load the samples or the presence of phytochemicals (Figure 5) [31,34].

#### 2.2.5. Morphological Structures of Synthesized AC-AuNPs

**Figure 6:**
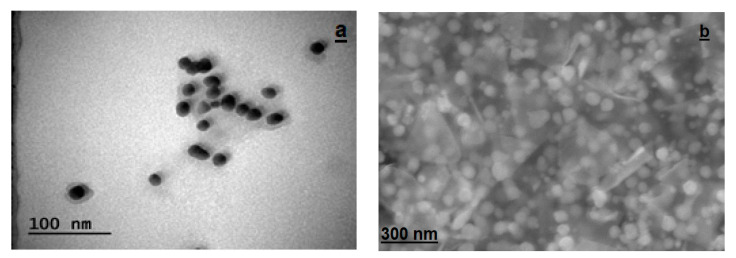
Morphological structures of the synthesized AC-AuNPs; (**a**) TEM, and (**b**) FESEM micrograph images.

TEM (transmission electron microscopy) and FESEM (field-emission scanning electron microscopy) techniques were used to determine the size, morphology, and dispersion of the biogenic gold nanoparticles. Figure 6a shows a TEM micrograph of the biogenic gold nanoparticles. It was observed that the prepared gold nanoparticles were almost spherical in shape. The nanoparticles were well separated from each other, suggesting a significant degree of stabilization by the phytochemicals in the aqueous extract of *Amygdalus communis* (Figure 6a). Similarly, Figure 6b shows an FESEM image of the biogenic gold nanoparticles.

#### 2.2.6. Surface Charge and Size Distributions of Synthesized AC-AuNPs

**Figure 7:**
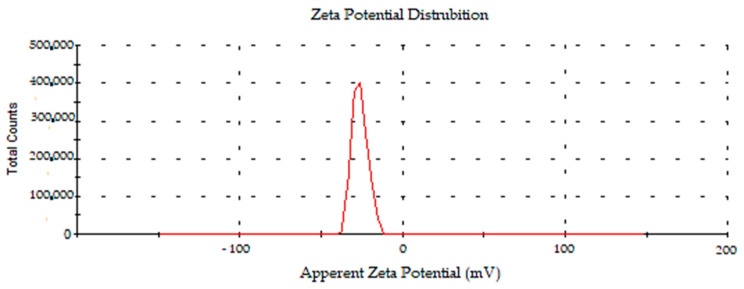
Zeta potential distribution of synthesized AC-AuNPs.

The zeta potential is a parameter that represents a particle’s charge and indicates the colloidal system’s potential stability. The zeta potential value is important for understanding and forecasting interactions between particles in suspension, and this notion is used in the study of cell adhesion, which is associated with surface charge features. The density-dependent surface charge and size distributions of the AC-AuNPs generated with AC leaf extract are shown in Figure 6 and Figure 7. The surface charge of the AC-AuNPs was found to be −27.7 ± 5.0 mV in the zeta potential measurement (Figure 7). Plant compounds actively contributed to the development of negative surface charges. The absence of conditions such as aggregation and fluctuation—both of which have detrimental impacts on stability—was made possible by the fact that the surface charge distribution was solely in the negative charge range. The negative surface charge also positively contributed to pH stability. In addition, the stability of the AuNP as a therapeutic agent is important for drug transport systems into the cell or in blood circulation for medical applications [35,36,37,38].

**Figure 8:**
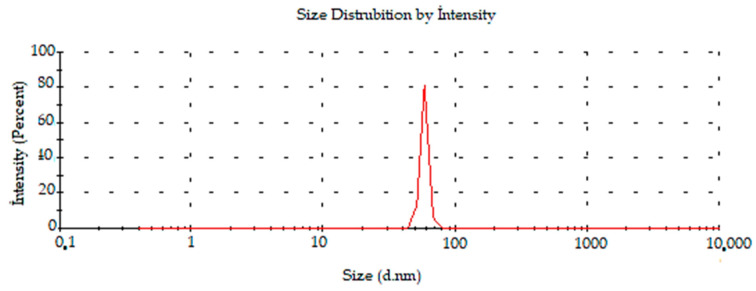
Distribution of density-dependent sizes of synthesized AC-AuNPs.

In previous experiments investigating the green synthesis of AuNPs, the surface charges of AuNPs synthesized with *Mimusops elengi* raw fruit extract, *Borassus flabellifer* fruit extract, and *Gracilaria verrucosa* extract were reported to be −22.0, −31.5, and −21.3 mV respectively [38,39,40]. It was determined by DLS that the average size distribution of the synthesized AC-AuNPs was 58.30 ± 3.6 d.nm, as shown in Figure 8.

#### 2.2.7. TGA-DTA Analysis Results of Synthesized AC-AuNPs

**Figure 9:**
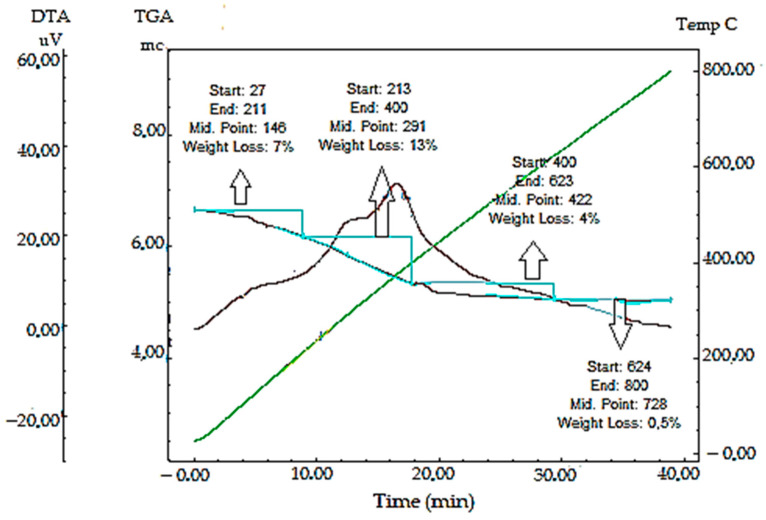
Mass loss points that occurred in the TGA-DTA data of synthesized AuNPs during temperature changes.

The resistance of the synthesized AC-AuNPs to temperature fluctuations was measured by assessing the findings of the TGA-DTA study performed in the 0–800 °C range. As a result of temperature variations, mass loss was detected at four separate sites. The first mass loss (7%) was thought to be produced by adsorbed water, while the second (13%), third (4%), and fourth (0.4%) mass losses were thought to be caused by bioorganic molecules, namely phytochemicals (Table 2, Figure 9) [41]. This demonstrated the existence of phytochemicals around the nanoparticles and that they were responsible for the stability due to the phytochemicals’ negative surface charge.

#### 2.2.8. AFM Analysis Results of Synthesized AC-AuNPs

**Figure 10:**
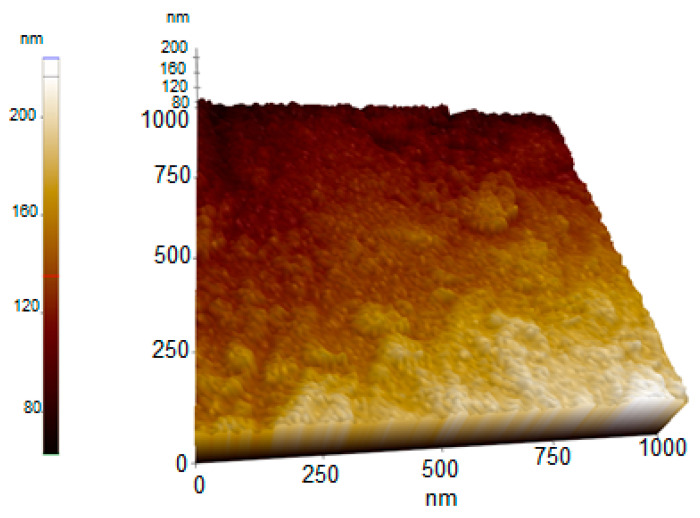
AFM micrograph of synthesized AC-AuNPs.

An AFM image demonstrating the shape, topographic, and size distributions of the synthesized AC-AuNPs is presented in Figure 10. It was observed that the AC-AuNPs had dimensions below 60 nm, a spherical appearance, and were monodisperse [42,43]. The size distribution of 58 nm shown in Figure 8 supported this finding. The fact that only a negative charge of −27.7 mV was detected, given in Figure 7, explained these results. In addition, the FESEM and TEM micrographs given in Figure 6 confirmed these findings for both the spherical morphological structure and single size distribution.

### 2.3. Biomedical Application of AC-AuNPs

#### 2.3.1. Antimicrobial Assay

**Figure 11:**
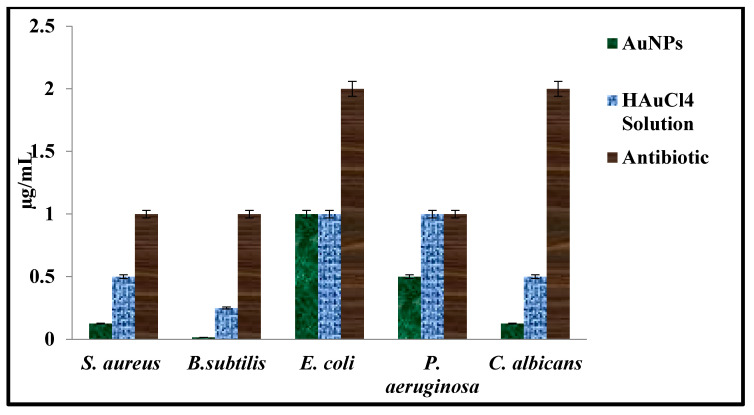
MIC concentrations of synthesized AC-AuNPs, HAuCI_4_ solution, and antibiotics (vancomycin, colistin, and fluconazole).

The antimicrobial suppressive effects of the AC-AuNPs, HAuCl_4_ solution, and standard antibiotics on human pathogen growth were evaluated by determining the MIC value using microdilution methods. It was determined that 0.50–1.00 µg mL^−1^ concentrations for gram-negative strains were effective MIC values for growth suppression. On the other hand, 0.02–0.12 and 0.12 µg mL^−1^ concentrations were determined as effective growth suppression concentrations for gram-positive strains, and *C. albicans* yeast, respectively. The AC-AuNPs had suppressive effects on the *E. coli* strain at lower concentrations than antibiotics and at the same concentration as the HAuCl_4_ solution. The AC-AuNPs were demonstrated to decrease the growth of other tested pathogens at extremely low concentrations compared to the standard antibiotics and HAuCl_4_ solution (Table 3, Figure 11).

The antibacterial impact of the AC-AuNPs at different doses is dependent on the strain’s cell wall thickness and different components, as well as the existence of a thinner peptidoglycan layer and lipopolysaccharide layer in the cell walls of gram-negative strains [32]. Some properties, such as concentration, size, surface charge, and shape, are very important to the antimicrobial effects of AuNPs. In aqueous medium, NPs interact with negatively charged microorganisms to return to their positively charged form, with the effect of electrostatic attraction [44,45].

After the interaction of AuNPs with microorganisms, adverse changes in wall and membrane morphology (such as disruption of membrane potential) occur. In addition, AuNPs cause irreversible damage to the cell wall by interacting with proteins where sulfur, nitrogen, phosphate, and oxygen atoms are concentrated. AuNPs block ATPase activity by preventing tRNA from binding to ribosomes, resulting in a decrease in ATP level [32]. NPs cause an increase in the expression of genes involved in redox reactions. They interact with important biomolecules such as proteins, lipids, DNA, and enzymes by increasing levels of reactive oxygen species (ROS) such as OH, SO, and NO. Then, they disrupt the microorganism’s structure and functions. As a result of these, AuNPs cause the death of microorganisms by accelerating their biological collapse [32,46,47].

The concentrations at which AuNPs obtained in different green synthesis studies suppressed the growth of microorganisms are given in detail in Table 4.

#### 2.3.2. Assessment of Cell Viability—MTT Assay

**Figure 12:**
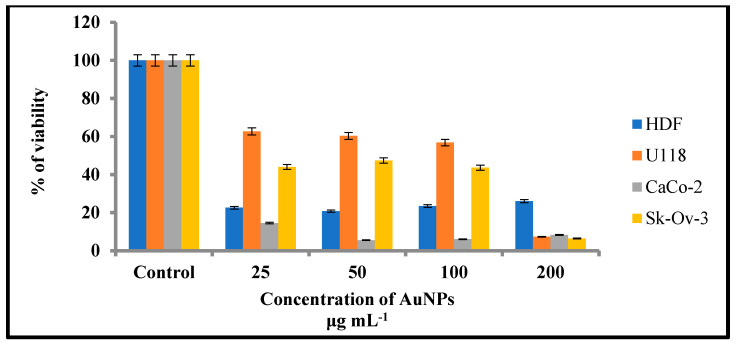
Cell viability rates after 48 h of interaction with AC-AuNPs applied at varying concentrations.

Gold nanoparticles have shown potential in cancer therapy. AuNPs offer several distinguishing characteristics, including tiny size, non-toxicity, and non-immunogenicity, which make them ideal candidates for targeted drug delivery systems. As tumor-targeting delivery vectors become smaller, the potential to evade the body’s natural barriers and impediments become more feasible. In the current study, the cytotoxic efficiency of the AC-AuNPs was tested against colorectal adenocarcinoma (Caco-2), glioblastoma (U118), and human ovarian sarcoma (Sk-ov-3) cancer cell lines and human dermal fibroblasts (HDF; healthy cells) using the MTT assay. The AC-AuNPs in the concentration range of 25–100 g mL^−1^ produced the greatest anticancer effects on CaCo-2 cells, as shown in Table 5 and Figure 12. This impact occurred at a rate of 85.49–94.49% viability suppression. The AC-AuNPs also had a substantial anticancer impact on Skov-3 and U118 cancer cell lines, decreasing their viability by 55.98–93.56% and 37.36–92.71%, respectively. Shape, size, concentration, surface charge, and contact duration are all important factors in nanoparticle toxicity [31]. Large pores (which facilitate the flow of chemicals such as nutrients and oxygen) can be seen in the vascular arteries of tumor and inflammatory tissues. Nanoparticles can easily aggregate and pass through these wide holes. Small AuNPs may easily pass through the cell membrane and produce effects such as generating ROS inside the cell and inducing apoptosis by activating caspase enzymes [52].

Table 6 shows the effective doses of AuNPs generated using the green synthesis approach on several cancer cell lines.

## 3. Materials and Methods

### 3.1. Materials

#### 3.1.1. Plant Materials

*A. communis* leaves were collected towards the end of May in the Mardin (Zinnar Valley) area. The leaves were rinsed with distilled water after being washed with tap water. A total of 10 g was added to a 250 mL beaker after drying at room temperature, to which 100 mL of distilled water was then added and brought to a boil. The mixture was filtered through paper after cooling and made suitable for synthesis.

#### 3.1.2. Standard and Reagents

The acetonitrile and methanol were gradient grade, while the formic acid and ammonium formate were liquid chromatography (LC) grade (analytical purity 99.9%), all of which were provided commercially from Sigma-Aldrich (Darmstadt, Germany). A 25 millimolar (mM) metal solution was prepared with the solid compound form of Alfa Aesar tetrachloroauric (III) acid (hydrogen tetrachloroaurate(III) trihydrate, ACS, 99.99% (metals basis), Au 49.0% min, CAS: 16961-25-4; (Kandel, Germany) to be used in the synthesis of the AC-AuNPs.

### 3.2. Methods

#### 3.2.1. Determination of Phenolic Compounds by LC-ESI-MS/MS

A 100 mg sample of dry plant extract was mixed with 10 mL of methanol and the solution was diluted to 2 mg/mL with 50% methanol in high-purity water. Afterward, the solution was filtered through a 0.22 mm filter and transferred to a vial prior to LC-ESI/MS/MS analysis. A Poroshell 120 EC-C18 column (100 mm 4.6 mm I.D., 2.7 mm) was used to chromatographically separate the components. The filtered plant mixture was passed down the column using a 0.1% formic acid carrier phase and 5 mM ammonium formate mobile phase, as well as mobile phase B consisting of 0.1% formic acid in methanol and 5 mM ammonium formate [57].

#### 3.2.2. Synthesis and Characterization of AuNPs

A 30 mL sample of the prepared leaf extract and 120 mL of 25 mM tetrachloroauric (III) acid solution (HAuCl_4_ solution) were mixed in a 1:4 ratio. After mixing the extract and metal solution, the reaction was observed at 10, 15, 30, 45, and 60 min. To confirm the presence of AuNPs, measurements were made in the range of 300–800 nm using a Perkin Elmer One UV-visible spectrophotometer. The elemental composition of the synthesized particles was determined by RadB-DMAX II computer-controlled Electron Disperse X-ray (EDX). The crystal structures of the AC-AuNPs obtained as a result of the synthesis were explained by the measurements obtained in the range of 10–80° at 2θ using the Rigaku Miniflex 600 model X-Ray Diffraction Diffractometer (XRD). The crystal nano dimension calculations were made using the Debye-Scherer equation given below [31].

D = Kλ/(β cosθ)(1)

where D is the grain size of the particle (nm), k is the Scherer constant (k = 0.94), λ is the X-ray wavelength, β is the full width at half maximum (FWHM) of the diffraction peak, and θ is the diffraction angle.

Micrographs obtained using the Park System XE-100 Atomic Power Microscope (AFM), Jeol Jem 1010 Transmission Electron Microscope (TEM), and Quanta FEG Field Emission Scan Electron Microscope (FE-SEM) were used to determine the morphological structure and appearance of the produced AC-AuNPs. Zeta potential measurements were performed using a Zetasizer Nano NS (Malvern, Uk) at pH 2–12, which had a significant impact on stability. Thermogravimetric and differential thermal analysis (TGA-DTA) was performed using the Shimadzu TGA-50 to assess the temperature resistance of the AC-AuNPs in the range of 0–800 °C. Fourier transform infrared spectroscopy (FTIR) was performed using the PerkinElmer One to evaluate the functional groups responsible for the bioreduction of the synthesized AuNPs in the range of 4000–650 cm^−1^.

#### 3.2.3. Determination of Growth Suppression Effects of AC-AuNPs against Pathogenic Microorganisms

By determining the minimum inhibition concentration (MIC) values using the microdilution technique, the inhibitory effects of the AC-AuNPs on the growth of human pathogens were identified [31,34]. The inhibitory effects of the AC-AuNPs were tested against gram-positive (*Staphylococcus aureus (S. aureus*) ATCC 29213; *Bacillus subtilis* (*B. subtilis*) ATCC 11774) and gram-negative (*Escherichia coli* (*E. coli*) ATCC 25922; *Pseudomonas aeruginosa* (*P. aeruginosa*) ATCC27833) bacterial strains and *Candida albicans* (*C. albicans*) yeast. The bacterial strains and yeast were obtained from Mardin Artuklu University Microbiology Research Laboratory and experimental studies were carried out. Furthermore, the effects of standard antibiotics and HAuCl_4_ solution on the pathogenic strains were examined using the microdilution technique. Fluconazole was used for *C. albicans*, and colistin and vancomycin antibiotics were used for gram-negative and -positive bacteria, respectively, as standard antibiotics. The CLSI M07-A8 standard broth dilution technique was used to test the antibacterial and antifungal effectiveness of gold nanoparticles by observing how quickly the microbes grew in the agar broth. We employed modified bacterial and fungal concentrations (108 CFU/mL, 0.5 McFarland’s standard) together with serial two-fold dilutions of gold nanoparticles in concentrations ranging from 5 to 0.156 mg/mL to calculate the MIC in BHI broth [55,56]. The control was incubated for 24 h at 37 °C and solely included inoculated broth. The MIC endpoint was the lowest gold nanoparticle concentration at which there was no discernible growth in the tubes. Before and after incubation, the visual turbidity of the tubes was documented to confirm the MIC value.

#### 3.2.4. Determination of Anticancer Effects of AC-AuNPs

The MTT (3-(4,5-Dimethylthiazol-2-yl)-2,5-diphenyltetrazolium bromide) assay was used to investigate the impact of the produced AC-AuNPs on cells in vitro. Human dermal fibroblast (HDF) healthy cells and colorectal adenocarcinoma (Caco-2), glioblastoma (U118), and human ovarian sarcoma (Sk-ov-3) cancer cells were acquired from Dicle University Scientific Research Center (DUBAM), Cell Culture Laboratory, Diyarbakir, Turkey. Sk-ov-3 cells were cultured in RPMI medium, whereas HDF, Caco-2, and U118 cells were grown in DMEM medium (Dulbecco’s Modified Eagle Medium). All cell lines were cultured in a humidified incubator at 37 °C with 95% air and 5% CO_2_. At the end of the period, the hemocytometer-controlled cells were added to 96-well microplates and incubated overnight. The AC-AuNPs in varying concentrations were added to the microplate wells containing cells and allowed to interact for 48 h in a CO_2_ incubator at 37 °C. Subsequently, the absorbance was measured at 540 nm using a MultiSkan Go (Thermo Fisher Scientific). The concentration at which cell viability was decreased upon contact with the AC-AuNPs was calculated using the method below [34].

% viability = U/C × 10(2)

where U is the absorbance value of AuNP-applied cells, and C is the absorbance value of control cells (without AuNPs in the medium).

## 4. Conclusions

The remarkable features of AuNPs create large application opportunities in biomedicine, making these compounds extremely desirable. In this green synthesis investigation, AC-AuNPs were synthesized in a quick, simple, low-cost, and environmentally friendly manner utilizing an extract derived from *Amygdalus communis* leaves, which are considered agricultural waste. UV-Vis, zeta potential, XRD, FESEM, TEM, AFM, TGA-DTA, and EDX techniques were used to investigate the characteristics of the AC-AuNPs. The AC-AuNPs showed a maximum absorbance at 533 nm, single size distribution, and stable structure, with a surface charge of −27.7 mV. The nanoparticles were spherical with a size in the 11–15 nm range, according to the TEM examination. The crystalline characteristic of the AuNPs was determined by the XRD pattern. The EDX spectrum showed a significant band of approximately 2–2.5 keV, indicating that elemental gold was a significant constituent of the gold nanoparticles. The nanoparticles were well dispersed through the sample, as shown in the solid phase. It seems that the aqueous extract of *A. communis* leaves prevented the aggregation of AuNPs. The microdilution technique was used to evaluate the effects of the AC-AuNPs on human pathogens in a bid to determine their usability as a therapeutic agent. The AC-AuNPs were shown to exhibit antibacterial and antifungal effects, significantly suppressing microorganism growth at concentrations ranging from 0.02–1.00 g/mL. The MTT technique was used to test the suppressive effects of the AC-AuNPs on the vitality of cancer cells, and it was found that they had excellent anticancer effects, particularly against Caco-2 cancer cells, with a viability suppression rate of 94.49%. It was also determined that the AC-AuNPs had a substantial suppressive impact on other cancer cells. Previous research has shown that several herbs have a wide range of actions, such as those involved in pathways that may become unbalanced during the evolution of malignancies. Thus, some herbs may be capable of suppressing malignancies and may be used as chemopreventive drugs. Currently, researchers are more interested in using these plants to counteract toxicity. The chemical composition of the *A. communis* plant reveals that it is an important resource for this type of research. Additionally, improving or modifying the properties of the synthesized AC-AuNPs will significantly contribute to studies on cancer (human colorectal adenocarcinoma, Caco-2) and antibiotic resistance problems in diseases of bacterial origin.

## Figures and Tables

**Table 1:** LC-MS/MS quantitation results of *Amygdalus communis* methanol extract.

Number	Standard	(RT)	R^2^	RSD	MI (m/z)	Linearity Range (μg/mL)	LOD (μg/mL)	LOQ (μg/mL)	Recovery (%)	*Amygdalus communis* Final Conc (ng/mL)
1	Shikimic acid	1.20	0.99	1.92	159.2	0.1–5	12.1	16.2	99.80	18,034.30
2	Gallic acid	1.70	0.99	1.58	168.9	0.1–5	13.2	17.0	100.10	1213.10
3	Protocatechuic acid	2.70	0.96	1.39	152.8	0.1–5	21.9	38.6	99.72	425.70
4	Gentisic acid	2.30	0.99	1.84	152.7	0.1–5	18.5	28.2	99.60	ND
5	Catechin	4.10	0.99	2.10	288.6	0.2–10	55.0	78.0	100.20	178.10
6	4-Hydroxybenzoic acid	8.70	0.99	1.20	137.3	0.2–10	68.4	88.1	100.30	ND
7	Chlorogenic acid	7.50	0.99	2.10	353.1	0.1–5	13.1	17.6	100.00	ND
8	4-Hydroxybenzaldehyde	5.80	0.99	2.20	121.9	0.1–5	20.1	36.1	100.00	ND
9	Vanillic acid	7.80	0.99	1.90	107.9	1–50	141.9	164.9	100.20	ND
10	Caffeic Acid	6.10	0.99	1.10	178.8	0.05–2.5	7.7	9.5	100.20	ND
11	Epicatechin	4.10	0.99	1.5	288.8	1–50	139.6	161.6	100.10	ND
12	Syringic acid	8.40	0.99	1.20	197.1	1–50	82.3	104.5	100.10	ND
13	P-coumaric acid	8.50	0.99	1.90	162.9	0.1–5	25.9	34.9	100.50	162.40
14	Salicylic Acid	8.70	0.99	1.40	136.9	0.1–5	6.0	8.3	99.90	694.80
15	Taxifolin	9.10	0.99	1.50	199.2	0.1–5	9.2	12.1	99.90	ND
16	Polydatine	9.30	0.99	1.40	182.8	0.1–5	12.1	19.2	100.00	315.60
17	Trans-ferulic acid	9.50	0.99	1.40	196.3	1–50	11.8	15.6	99.50	ND
18	Sinapic acid	11.40	0.99	1.45	222.9	0.2–10	65.2	82.3	99.90	ND
19	Quercimeritrin	10.7	0.99	1.90	137.9	0.1–5	68.5	88.2	99.60	2015.4
20	Coumarin	10.6	0.99	2.20	152.3	0.1–5	214.2	247.3	99.50	ND
21	Scutellarin	12.9	0.99	1.30	198.3	0.1–5	16.2	21.2	99.50	ND
22	O-coumaric acid	8.5	0.99	2.10	163.0	0.1–5	31.8	40.4	100.00	ND
23	Cynarin	11.4	0.99	1.55	289.3	0.1–5	19.5	28.5	100.00	ND
24	Protocatechuic ethyl ester	11.2	0.99	1.45	137.2	0.1–5	15.4	22.2	100.00	ND
25	Hyperoside	11.6	0.99	1.80	289.1	0.1–5	139.5	161.5	99.90	1773.1
26	Quercetin-3-glucoside	11.9	0.99	1.75	304.1	0.1–5	4.9	6.5	100.00	2448.0
27	Rutin	11.9	0.99	2.10	610.2	0.1–5	15.9	22.9	99.80	16,564.6
28	Resveratrol	11.7	0.99	1.10	301.1	0.1–5	7.1	9.1	100.00	ND
29	Naringin	11.1.	0.99	1.25	269.8	0.1–5	2.6	3.9	100.60	ND
30	Rosmarinic acid	12.5	0.99	1.55	360.1	0.1–5	16.2	21.2	100.60	ND
31	Quercetin-3-D-xyloside	12.5	0.99	1.65	305.2	0.1–5	N.A	N.A	100.10	ND
32	Hesperidin	12.5	0.99	1.60	450.1	0.1–5	19.0	26.0	99.70	ND
33	Kaemerol-3-glucoside	13.3	0.99	1.35	285.1	0.1–5	10.4	15.6	99.90	53.2
34	Fisetin	13.4	0.99	1.25	285.0	0.1–5	10.1	12.7	99.80	ND
35	Oleuropein	13.9	0.99	1.20	540.2	0.1–5	24.6	30.6	99.90	ND
36	Baicalin	13.8	0.99	1.25	154.2	0.1–5	24.3	30.2	99.90	ND
37	Trans-cinnamic acid	14.3	0.99	1.30	149.8	0.1–5	215.1	240.2	99.90	ND
38	Ellagic acid	15.0	0.99	1.50	302.1	0.1–5	56.9	71.1	100.10	ND
39	Quercetin	15.0	0.99	1.65	301.1	0.1–5	15.5	19.0	99.70	298.7
40	Naringenin	15.1	0.99	2.35	270.8	0.1–5	2.6	3.9	100.60	66.3
41	Silibinin	15.8	0.99	2.20	440.1	0.1–5	19.3	28.3	99.90	ND
42	Hesperetin	15.4	0.99	2.50	301.2	0.1–5	7.1	9.1	100.00	ND
43	Morin	13.2	0.99	2.10	152.9	0.1–5	22.3	28.4	100.00	ND
44	Kaempferol	19.5	0.99	1.85	286.1	0.1–5	10.2	15.4	99.90	ND
45	Tamarixetin	17.5	0.99	1.90	163.1	0.1–5	25.8	34.8	99.90	ND
46	Baicalein	17.6	0.99	1.75	158.2	0.1–5	23.9	32.7	99.90	ND
47	7-Hydroxyflavone	18.6	0.99	1.65	222.2	0.1–5	64.9	82.1	99.90	ND
48	6-Hydroxyflavone	19.8	0.99	1.55	138.1	0.1–5	5.9	8.2	100.00	ND
49	Biochanin A	20.5	0.99	1.20	145.2	0.1–5	212.4	244.2	99.80	89.5
50	Chrysin	20.8	0.99	1.55	147.1	0.1–5	0.012	0.012	99.90	ND
51	5-Hydroxyflavone	23.9	0.99	1.65	252.7	0.1–5	9.7	11.5	100.00	ND
52	6,2,4-Trimetoxyflavone	24.9	0.99	1.70	585.2	0.1–5	11.1	15.5	99.90	ND
53	Diosgenin	30.5	0.99	1.10	285.5	0.1–5	11.8	16.6	100.00	36.9

Abbreviations: LC-MS/MS; Liquid chromatography-tandem mass spectrometry; LOD: Limit of detection; LOQ: Limit of quantification; N.A: No answer; ND: Not determined; RSD: Relative standard deviation; RT: Retention time.

**Table 2:** Temperature points where mass losses occurred against the resistance of synthesized AC-AuNPs to heat treatment.

Mass Loss Point	Temperature (°C)	Mass Loss (%)
First	27–211	7
Second	213–400	13
Third	400–623	4
Fourth	624–800	0.5

**Table 3:** MIC values of synthesized AC-AuNPs.

Tested Microorganism	AuNPs µg mL^−1^	HAuCI_4_ Solution µg mL^−1^	* Antibiotics µg mL^−1^
*E. coli*	1.00	1.00	2.00
*P. aeruginosa*	0.50	1.00	1.00
*S. aureus*	0.12	0.50	1.00
*B. subtilis*	0.02	0.25	1.00
*C. albicans*	0.12	0.50	2.00

* Antibiotics: colistin (gram-positive bacteria), vancomycin (gram-negative bacteria), and fluconazole (*C. albicans* yeast).

**Table 4:** The concentrations of AuNPs that were effective in suppressing the growth of microorganisms in different biologically sourced green synthesis studies.

MIC Values of AuNPs µg mL^−1^				
Green Synthesis Source	Gram Positive *S.aureus*/*B. subtilis*	Gram Negative *E.coli*/*P. aeruginosa*	*C. albicans*	Ref.
*Cydonia oblonga*	0.15	0.05	0.13	[41]
*Crataegus monogyna*	0.05/0.02	0.50/0.25	0.11	[48]
*Prunus cerasifera*	0.25/0.12	1.00/0.50	0.50	[49]
*Jatropha integerrima Jacq.*	10.0/5.00	2.5	-	[50]
*Zingiber officinale*	30	-	-	[51]

**Table 5:** Percent (%) viability rates of cell lines after interaction with varying concentration ranges of AC-AuNPs.

Cell Lines	Concentrations (µg mL^−1^)			
	25	50	100	200
HDF	22.6 *	20.8	23.5	26.1
U118	62.6	60.3	56.8	7.3
Caco-2	14.5	5.5	6.0	8.3
Sk-ov-3	44.0	47.4	43.6	6.4

* % viability.

**Table 6:** Comparison of the % viability-suppressing effect concentrations of some green-synthesized AuNPs on different cancer cell lines.

Green Synthesis Source	Cell Line	Dimension (nm)	Shape	Effective Concentration (µg mL^−1^)	Ref.
*H. spinosa*	Sk-ov-3	68.4	Spherical	47.5	[28]
*M. indica*	MCF-7	19.5	Spherical	400	[32]
*G. tournefortii*	U118	5–10	Spherical	100	[53]
*H. sabdariffa*	U87	30	Spherical	2.5	[54]
*B. verna*	HeLa	11	Spherical	2	[55]
*C. baccata*	Caco-2	8.4	Spherical	400	[56]
*A. communis*	Caco-2, Sk-ov-3, U118	58	Spherical	25	This study

## Data Availability

All data used to support the findings of this study are included in the article.

## Sample Availability

Samples of the compounds of AC-AuNPs are available from the corresponding authors.

## References

[1] Nejati K., Dadashpour M., Gharibi T., Mellatyar H., Akbarzadeh A. (2021). Biomedical applications of functionalized gold nanoparticles: A review. J. Clust. Sci..

[2] Kwon H.J., Shin K., Soh M., Chang H., Kim J., Lee J., Ko G., Kim B.H., Kim D., Hyeon T. (2018). Large-scale synthesis and medical applications of uniform-sized metal oxide nanoparticles. Adv. Mater..

[3] Akintelu S.A., Yao B., Folorunso A.S. (2021). Green synthesis, characterization, and antibacterial investigation of synthesized gold nanoparticles (AuNPs) from *Garcinia kola* pulp extract. Plasmonics.

[4] Giljohann D.A., Seferos D.S., Daniel W.L., Massich M.D., Patel P.C., Mirkin C.A. (2020). Gold nanoparticles for biology and medicine.

[5] Dou X., Wang X., Qian S., Liu N., Yuan X. (2020). From understanding the roles of tetraoctylammonium bromide in the two-phase Brust–Schiffrin method to tuning the size of gold nanoclusters. Nanoscale.

[6] Canaparo R., Foglietta F., Limongi T., Serpe L. (2020). Biomedical applications of reactive oxygen species generation by metal nanoparticles. Materials.

[7] Rehman A., John P., Bhatti A. (2021). Biogenic selenium nanoparticles: Potential solution to oxidative stress mediated inflammation in rheumatoid arthritis and associated complications. J. Nanomater..

[8] Singh P., Mijakovic I. (2021). Advances in gold nanoparticle technology as a tool for diagnostics and treatment of cancer. Expert Rev. Mol. Diagn..

[9] Bucharskaya A.B., Khlebtsov N.G., Khlebtsov B.N., Maslyakova G.N., Navolokin N.A., Genin V.D., Genina E.A., Tuchin V.V. (2022). Photothermal and photodynamic therapy of tumors with plasmonic nanoparticles: Challenges and prospects. Materials.

[10] Ferreira D., Fontinha D., Martins C., Pires D., Fernandes A.R., Baptista P.V. (2020). Gold nanoparticles for vectorization of nucleic acids for cancer therapeutics. Molecules.

[11] Ye J., Wen Q., Wu Y., Fu Q., Zhang X., Wang J., Gao S., Song J. (2022). Plasmonic anisotropic gold nanorods: Preparation and biomedical applications. Nano Res..

[12] Yang W., Liang H., Ma S., Wang D., Huang J. (2019). Gold nanoparticle based photothermal therapy: Development and application for effective cancer treatment. Sustain. Mater. Technol..

[13] Jamkhande P.G., Ghule N.W., Bamer A.H., Kalaskar M.G. (2019). Metal nanoparticles synthesis: An overview on methods of preparation, advantages and disadvantages, and applications. J. Drug Deliv. Sci. Technol..

[14] Saratale R.G., Saratale G.D., Shin H.S., Jacob J.M., Pugazhendhi A., Bhaisare M., Kumar G. (2018). New insights on the green synthesis of metallic nanoparticles using plant and waste biomaterials: Current knowledge, their agricultural and environmental applications. Environ. Sci. Pollut. Res..

[15] Nawade B., Yahyaa M., Reuveny H., Shaltiel-Harpaz L., Eisenbach O., Faigenboim A., Holland D., Ibdah M. (2019). Profiling of volatile terpenes from almond (*Prunus dulcis*) young fruits and characterization of seven terpene synthase genes. Plant Sci..

[16] Özcan M.M., Al Juhaimi F., Ghafoor K., Babiker E.E., Özcan M.M. (2020). Characterization of physico-chemical and bioactive properties of oils of some important almond cultivars by cold press and soxhlet extraction. J. Food Sci. Technol..

[17] Tlili N., Kirkan B., Sarikurkcu C. (2019). LC–ESI–MS/MS characterization, antioxidant power and inhibitory effects on α-amylase and tyrosinase of bioactive compounds from hulls of *Amygdalus communis*: The influence of the extracting solvents. Ind. Crops Prod..

[18] Farhadi S., Javanmard M., Safavi M. (2022). Sour-Cherry Seed Polyphenol Contents, Antioxidant Activity and Nutritional Components as a Potential Bioactive Source. Nutr. Food Sci. Res..

[19] Sahiba N., Sethiya A.K., Agarwal D., Agarwal S. (2022). An Overview on Immunity Booster Foods in Coronavirus Disease (COVID-19). Comb. Chem. High Throughput Screen..

[20] Fraihat A., Hamdan F.R., Abu-Irmaileh B., Abbasi R., Abu-Irmaileh B., Bustanji Y. (2018). Evaluation of the antiproliferative Activities of *Anthemis bornmuelleri* L. and *Amygdalis communis* L. Extracts Against six Human Cancer cell lines. Res. J. Pharm. Technol..

[21] Kahlaoui M., Borotto Dalla Vecchia S., Giovine F., Ben Haj Kbaier H., Bouzouita N., Barbosa Pereira L., Zeppa G. (2019). Characterization of polyphenolic compounds extracted from different varieties of almond hulls (*Prunus dulcis* L.). Antioxidants.

[22] Barral-Martinez M., Fraga-Corral M., Garcia-Perez P., Simal-Gandara J., Prieto M.A. (2021). Almond by-products: Valorization for sustainability and competitiveness of the industry. Foods.

[23] Sheng Q., Yi L., Zhong B., Wu X., Liu L., Zhang B. (2023). Shikimic acid biosynthesis in microorganisms: Current status and future direction. Biotechnol. Adv..

[24] Karami A., Fakhri S., Kooshki L., Khan H. (2022). Polydatin: Pharmacological Mechanisms, Therapeutic Targets, Biological Activities, and Health Benefits. Molecules.

[25] Luo J.L., Chen S., Wang L., Zhao X.H., Piao C.L. (2022). Pharmacological effects of polydatin in the treatment of metabolic diseases: A review. Phytomedicine.

[26] Satari A., Ghasemi S., Habtemariam S., Asgharian S., Lorigooini Z. (2021). Rutin: A flavonoid as an effective sensitizer for anticancer therapy; insights into multifaceted mechanisms and applicability for combination therapy. Evid.-Based Complement. Alternat. Med..

[27] Younis F.A., Ahmed H.A., Ahmed F.M., Awad R.M., Gibril M. (2018). Green Synthesis and Characterization of Gold Nanoparticles (AuNPs) Using Fenugreek Seeds Extract (*Trigonella foenum-graecum*). Eur. J. Pharm. Sci..

[28] Satpathy S., Patra A., Ahirwar B., Hussain M.D. (2020). Process optimization for green synthesis of gold nanoparticles mediated by extract of *Hygrophila spinosa* T. Anders and their biological applications. Phys. E Low-Dimens. Syst. Nanostructures.

[29] Vahidi H., Kobarfard F., Kosar Z., Mahjoub M.A., Saravanan M., Barabadi H. (2020). Mycosynthesis and characterization of selenium nanoparticles using standard penicillium chrysogenum PTCC 5031 and their antibacterial activity: A novel approach in microbial nanotechnology. Nanomed. J..

[30] Kumar P.S., Jeyalatha M.V., Malathi J., Ignacimuthu S. (2018). Anticancer effects of one-pot synthesized biogenic gold nanoparticles (Mc-AuNPs) against laryngeal carcinoma. J. Drug Deliv. Sci. Technol..

[31] Keskin C., Atalar M.N., Firat Baran M., Baran A. (2021). Environmentally friendly rapid synthesis of gold nanoparticles from *Artemisia absinthium* plant extract and application of antimicrobial activities. J. Inst. Sci. Technol..

[32] Donga S., Bhadu G.R., Chanda S. (2020). Antimicrobial, antioxidant and anticancer activities of gold nanoparticles green synthesized using *Mangifera indica* seed aqueous extract. Artif. Cells Nanomed. Biotechnol..

[33] Awad M.A., Eisa N.E., Virk P., Hendi A.A., Ortashi K.M., Mahgoub A.S., Eissa F.Z. (2019). Green synthesis of gold nanoparticles: Preparation, characterization, cytotoxicity, and anti-bacterial activities. Mater. Lett..

[34] Baran A., Baran M.F., Keskin C., Kandemir S.I., Valiyeva M., Mehraliyeva S., Khalilov R., Eftekhari A. (2021). Ecofriendly/rapid synthesis of silver nanoparticles using extract of waste parts of artichoke (*Cynara scolymus* L.) and evaluation of their cytotoxic and antibacterial activities. J. Nanomater..

[35] Nadhe S.B., Wadhwani S.A., Singh R., Chopade B.A. (2020). Green synthesis of AuNPs by Acinetobacter sp. GWRVA25: Optimization, characterization, and its antioxidant activity. Front. Chem..

[36] Khan A.U., Khan M., Malik N., Cho M.H., Khan M.M. (2019). Recent progress of algae and blue–green algae-assisted synthesis of gold nanoparticles for various applications. Bioprocess Biosyst. Eng..

[37] Lee K.X., Shameli K., Yew Y.P., Teow S.Y., Jahangirian H., Rafiee-Moghaddam R., Webster T.J. (2020). Recent developments in the facile bio-synthesis of gold nanoparticles (AuNPs) and their biomedical applications. Int. J. Nanomed..

[38] Tripathy A., Behera M., Rout A.S., Biswal S.K., Phule A.D. (2020). Optical, structural, and antimicrobial study of gold nanoparticles synthesized using an aqueous extract of mimusops elengi raw fruits. Biointerface Res. Appl. Chem..

[39] Chinnaiyan S.K., Soloman A.M., Perumal R.K., Gopinath A., Balaraman M. (2019). 5 Fluorouracil-loaded biosynthesised gold nanoparticles for the in vitro treatment of human pancreatic cancer cell. IET Nanobiotechnol..

[40] Chellapandian C., Ramkumar B., Puja P., Shanmuganathan R., Pugazhendhi A., Kumar P. (2019). Gold nanoparticles using red seaweed *Gracilaria verrucosa*: Green synthesis, characterization and biocompatibility studies. Process Biochem..

[41] Baran M.F. (2019). Synthesis, characterization and investigation of antimicrobial activity of silver nanoparticles from *Cydonia oblonga* leaf. Appl. Ecol. Environ. Res..

[42] Hatipoğlu A. (2021). Rapid green synthesis of gold nanoparticles: Synthesis, characterization and antimicrobial activities. Prog. Nutr..

[43] Nirala N.R., Prakash R. (2018). One step synthesis of AuNPs@ MoS2-QDs composite as a robust peroxidase-mimetic for instant unaided eye detection of glucose in serum, saliva and tear. Sens. Actuators B Chem..

[44] Rauf A., Ahmad T., Khan A., Maryam, Uddin G., Ahmad B., Mabkhot Y.N., Bawazeer S., Riaz N., Malikovna B.K. (2021). Green synthesis and biomedicinal applications of silver and gold nanoparticles functionalized with methanolic extract of *Mentha longifolia*. Artif. Cells Nanomed. Biotechnol..

[45] Francis S., Joseph S., Koshy E.P., Mathew B. (2017). Green synthesis and characterization of gold and silver nanoparticles using *Mussaenda glabrata* leaf extract and their environmental applications to dye degradation. Environ. Sci. Pollut. Res..

[46] Cui Y., Zhao Y., Tian Y., Zhang W., Lü X., Jiang X. (2012). The molecular mechanism of action of bactericidal gold nanoparticles on Escherichia coli. Biomaterials.

[47] Ahmed K.B.A., Raman T., Veerappan A. (2016). Future prospects of antibacterial metal nanoparticles as enzyme inhibitor. Mater. Sci. Eng. C.

[48] Baran A., Hatipoğlu A., Firat Baran M., Aktepe N. (2021). Evaluation of Synthesis and Antimicrobial Activities of Gold Nanoparticles from Hawthorn (*Crataegus monogyna*) Fruit Extract. Eur. J. Sci. Technol..

[49] Hatipoğlu A. (2021). Green synthesis of gold nanoparticles from *Prunus cerasifera pissardii nigra* leaf and their antimicrobial activities on some food pathogens. Prog. Nutr..

[50] Suriyakala G., Sathiyaraj S., Babujanarthanam R., Alarjani K.M., Hussein D.S., Rasheed R.A., Kanimozhi K. (2022). Green synthesis of gold nanoparticles using *Jatropha integerrima* Jacq. flower extract and their antibacterial activity. J. King Saud. Univ. Sci..

[51] Velmurugan P., Anbalagan K., Manosathyadevan M., Lee K.J., Cho M., Lee S.M., Oh B. (2014). Green synthesis of silver and gold nanoparticles using Zingiber officinale root extract and antibacterial activity of silver nanoparticles against food pathogens. Bioprocess Biosyst. Eng..

[52] Ramachandran R., Krishnaraj C., Sivakumar A.S., Prasannakumar P., Kumar V.A., Shim K.S., Yun S.I. (2017). Anticancer activity of biologically synthesized silver and gold nanoparticles on mouse myoblast cancer cells and their toxicity against embryonic zebrafish. Mater. Sci. Eng. C.

[53] Keskin C., Baran A., Baran M.F., Hatipoğlu A., Adican M.T., Atalar M.N., Huseynova I., Khalilov R., Ahmadian E., Yavuz Ö. (2022). Green Synthesis, Characterization of Gold Nanomaterials using *Gundelia tournefortii* Leaf Extract, and Determination of Their Nanomedicinal (Antibacterial, Antifungal, and Cytotoxic) Potential. J. Nanomater..

[54] Mishra P., Ray S., Sinha S., Das B., Khan M.I., Behera S.K., Mishra A. (2016). Facile bio-synthesis of gold nanoparticles by using extract of Hibiscus sabdariffa and evaluation of its cytotoxicity against U87 glioblastoma cells under hyperglycemic condition. Biochem. Eng. J..

[55] Hutchinson N., Wu Y., Wang Y., Kanungo M., DeBruine A., Kroll E., Gilmore D., Eckrose Z., Gaston S., Matel P. (2021). Green Synthesis of Gold Nanoparticles Using Upland Cress and Their Biochemical Characterization and Assessment. J. Nanomater..

[56] González-Ballesteros N., Prado-López S., Rodríguez-González J.B., Lastra M., Rodríguez-Argüelles M.C. (2017). Green synthesis of gold nanoparticles using brown algae *Cystoseira baccata*: Its activity in colon cancer cells. Colloids Surf. B Biointerfaces.

[57] Oniszczuk A., Olech M., Oniszczuk T., Wojtunik-Kulesza K., Wójtowicz A. (2019). Extraction methods, LC-ESI-MS/MS analysis of phenolic compounds and antiradical properties of functional food enriched with elderberry flowers or fruits. Arab. J. Chem..

